# Supplementary material for: The effects of genital myiasis on the diversity of the vaginal microbiota in female Bactrian camels
Source: BMC Vet Res. 2022 Mar 5;18:87. doi: 10.1186/s12917-022-03189-5 (PMC8897907; doi:10.1186/s12917-022-03189-5)
Supplement: Supplementary file 5 — Additional file 5. [file 12917_2022_3189_MOESM5_ESM.zip › MPL201709200_16s_yy/Treat1/css/js_css/jsPDF-master.zip!/jsPDF-master/docs/index.html]

Home - Documentation


## Home

### Classes

- jsPDF

### Global

- addFont
- addHTML
- addMetadata
- addPage
- autoPrint
- CapJoinStyles
- circle
- ellipse
- getFontList
- lines
- lstext
- output
- rect
- roundedRect
- save
- setDisplayMode
- setDrawColor
- setFillColor
- setFont
- setFontSize
- setFontStyle
- setLineCap
- setLineJoin
- setLineWidth
- setPage
- setProperties
- setTextColor
- text
- triangle

# jsPDF

**A library to generate PDFs in client-side JavaScript.**

You can catch me on twitter: @MrRio or head over to my company's website for consultancy.

## Live Demo | Documentation

## Creating your first document

The easiest way to get started is to drop the CDN hosted library into your page:

```
<script src="https://cdnjs.cloudflare.com/ajax/libs/jspdf/1.3.2/jspdf.debug.js"></script>
```

Then you're ready to start making your document:

```
// Default export is a4 paper, portrait, using milimeters for units
var doc = new jsPDF()

doc.text('Hello world!', 10, 10)
doc.save('a4.pdf')
```

If you want to change the paper size, orientation, or units, you can do:

```
// Landscape export, 2×4 inches
var doc = new jsPDF({
  orientation: 'landscape',
  unit: 'in',
  format: [4, 2]
})

doc.text('Hello world!', 10, 10)
doc.save('two-by-four.pdf')
```

Great! Now give us a Star :)

## Contributing

Build the library with `npm run build`. This will fetch all dependencies and then compile the `dist` files. To see the examples locally you can start a web server with `npm start` and go to `localhost:8000`.

## Credits

- Big thanks to Daniel Dotsenko from Willow Systems Corporation for making huge contributions to the codebase.
- Thanks to Ajaxian.com for featuring us back in 2009.
- Everyone else that's contributed patches or bug reports. You rock.

## License (MIT)

Copyright (c) 2010-2016 James Hall, https://github.com/MrRio/jsPDF

Permission is hereby granted, free of charge, to any person obtaining
a copy of this software and associated documentation files (the
"Software"), to deal in the Software without restriction, including
without limitation the rights to use, copy, modify, merge, publish,
distribute, sublicense, and/or sell copies of the Software, and to
permit persons to whom the Software is furnished to do so, subject to
the following conditions:

The above copyright notice and this permission notice shall be
included in all copies or substantial portions of the Software.

THE SOFTWARE IS PROVIDED "AS IS", WITHOUT WARRANTY OF ANY KIND,
EXPRESS OR IMPLIED, INCLUDING BUT NOT LIMITED TO THE WARRANTIES OF
MERCHANTABILITY, FITNESS FOR A PARTICULAR PURPOSE AND
NONINFRINGEMENT. IN NO EVENT SHALL THE AUTHORS OR COPYRIGHT HOLDERS BE
LIABLE FOR ANY CLAIM, DAMAGES OR OTHER LIABILITY, WHETHER IN AN ACTION
OF CONTRACT, TORT OR OTHERWISE, ARISING FROM, OUT OF OR IN CONNECTION
WITH THE SOFTWARE OR THE USE OR OTHER DEALINGS IN THE SOFTWARE.

  

Documentation generated by JSDoc 3.4.2 on Sun Oct 09 2016 11:08:27 GMT+0100 (BST) using the docdash theme.
